# The Role of Cardiovascular Risk Prediction Model Selection in Primary Prevention: An Observational Study of Statin Eligibility Agreement Across Nine Scores in a Lithuanian Primary-Prevention Cohort

**DOI:** 10.3390/medicina62050979

**Published:** 2026-05-17

**Authors:** Petras Navickas, Sigita Glaveckaitė, Laura Lukavičiūtė-Navickienė, Agnė Šatrauskienė, Arvydas Baranauskas, Egidija Rinkūnienė, Emilija Meškėnė, Vaida Šileikienė, Edita Lycholip, Aleksandras Laucevičius

**Affiliations:** 1 Institute of Clinical Medicine, Faculty of Medicine, Vilnius University, LT-03101 Vilnius, Lithuania; sigita.glaveckaite@santa.lt (S.G.); lukaviciute.laura@gmail.com (L.L.-N.); agne.satrauskiene@gmail.com (A.Š.); arvydas.baranauskas@santa.lt (A.B.); egidija.rinkuniene@santa.lt (E.R.); emilija.petrulionyte@santa.lt (E.M.); vaida.sileikiene@santa.lt (V.Š.); edita.lycholip@santa.lt (E.L.); 2 State Research Institute Centre for Innovative Medicine, LT-08406 Vilnius, Lithuania; aleksandras.laucevicius@santa.lt

**Keywords:** primary cardiovascular prevention, risk prediction models, statin eligibility, inter-model concordance

## Abstract

*Background and Objectives*: Cardiovascular risk prediction models (RPMs) are widely used to guide statin initiation in primary prevention, yet the extent to which different models produce concordant treatment decisions in the same population remains insufficiently characterized. We compared statin eligibility across nine commonly used RPMs: SCORE2, PREVENT, PCE, ASSIGN, FRS-hCHD, AusCVDRisk, MESA, QRISK3, and RRS. *Materials and Methods*: We performed a cross-sectional analysis of 11,174 adults aged 40–65 years with metabolic syndrome enrolled in the Lithuanian High Cardiovascular Risk primary prevention program (LitHiR) and evaluated them at a single tertiary center during 2006–2023. Statin eligibility was determined for each RPM using guideline-mapped treatment thresholds. Pairwise agreement was assessed using Cohen’s κ, Gwet’s AC1, Positive and Negative Percent Agreement (PPA/NPA), the Jaccard index, and McNemar testing. Analyses were repeated by sex. Consensus eligibility was defined as treatment recommended by at least k of nine models. *Results*: Eligibility varied more than twenty-fold, from 67.39% (7530/11,174) with SCORE2 to 3.03% (339/11,174) with AusCVDRisk; intermediate estimates included PREVENT at 44.83%, QRISK3 at 39.00%, and PCE at 37.97%. Overall pairwise agreement was modest: κ ranged from 0.03 (SCORE2 vs. AusCVDRisk) to 0.67 (QRISK3 vs. ASSIGN), with a median κ of 0.38 (IQR: 0.19–0.51). Median AC1 was 0.58 (IQR 0.37–0.68). Agreement was stronger for non-eligibility than for eligibility (median NPA: 0.82 vs. median PPA: 0.53). Consensus eligibility declined from 73.5% at k = 1 to 45.1% at k = 3, 30.0% at k = 5, and 1.87% at k = 9, with the greatest sex divergence at intermediate stringency. *Conclusions*: In this real-world cohort with elevated cardiometabolic risk, statin eligibility was highly dependent on RPM choice and showed only modest inter-model concordance. Increasing consensus stringency rapidly reduced eligibility, indicating that RPM selection and embedded thresholds substantially influence statin treatment decisions in primary prevention.

## 1. Introduction

Cardiovascular disease (CVD) remains the leading cause of premature morbidity and mortality worldwide, and a substantial proportion of events occur in individuals without established disease but with modifiable risk factors. A large body of evidence shows that lowering low-density lipoprotein cholesterol (LDL-C) levels reduces major vascular events across a broad range of baseline risks and therapeutic approaches, including statins and selected non-statin agents used in primary prevention (e.g., ezetimibe and PCSK9 inhibitors) [1]. In contemporary practice, however, lipid-lowering therapy is rarely initiated on the basis of isolated lipid values alone. Rather, absolute cardiovascular risk—usually expressed as a 5- or 10-year probability of a composite CVD outcome—serves as the main determinant of treatment recommendation, with thresholds defined in national and international guidelines [2,3].

To support risk-based decision-making, numerous cardiovascular risk prediction models (RPMs) have been developed and incorporated into primary prevention workflows. Commonly used examples include Systematic Coronary Risk Evaluation 2 (SCORE2) [4] in Europe, Pooled Cohort Equations (PCEs) [5] and the more recent Predicting Risk of cardiovascular disease EVENTs (PREVENT) [6,7] equation in the United States, QRISK3 [8] in the United Kingdom, Assessing Cardiovascular Risk using SIGN (ASSIGN) [9] in Scotland, the Australian cardiovascular disease risk score (AusCVDRisk) [10], and research-derived tools such as the Multi-Ethnic Study of Atherosclerosis (MESA) [11] and the Reynolds Risk Score (RRS) [12]. Across settings, clinicians are encouraged—or required—to initiate lipid-lowering therapy when estimated risk exceeds model-specific or guideline-specified thresholds, sometimes together with additional clinical qualifiers [2,3]. Consequently, RPM selection directly influences pharmacotherapy decisions in primary prevention.

A major challenge is that different RPMs often generate different eligibility classifications when applied to the same population, even when they are intended to predict broadly similar outcomes over similar time horizons. This discordance may arise from the differences in outcome definitions, predictor sets, model structure, regional recalibration, target populations, and treatment thresholds embedded in evolving guidelines [2]. Even modest variation in calibration or thresholding may shift substantial numbers of individuals above or below treatment cutoffs, thereby affecting projected benefits, resource allocation, and patient-level risk–benefit trade-offs [13].

These considerations raise two practical questions. First, how strongly do commonly used RPMs agree in determining eligibility for lipid-lowering therapy in a real-world cohort? Second, what is the operational impact of “consensus” approaches in which treatment is recommended only when several models concur? Although prior studies have examined the performance or calibration of individual models, fewer have systematically quantified inter-model agreement using complementary chance-corrected metrics and consensus-based decision rules [2]. This issue is not merely methodological; it has implications for clinical consistency, equitable access to preventive therapy, and policy decisions regarding which RPMs should be endorsed in specific populations.

Against this backdrop, we undertook a comprehensive, model-agnostic comparison of statin eligibility across nine widely used RPMs in a large, real-world cohort of middle-aged adults with elevated cardiometabolic risk enrolled in a structured primary-prevention program. Our objectives were to (i) estimate eligibility rates under each RPM using guideline-concordant thresholds; (ii) quantify pairwise agreement in eligibility using complementary metrics that distinguish overall agreement from agreement on positives vs. negatives; and (iii) characterize consensus footprints across increasing levels of cross-model agreement. By anchoring the analysis in routine clinical data and established calculators, this study aims to provide decision-relevant evidence on how the choice of RPM shapes statin eligibility classification in primary prevention, thereby informing clinicians, health-system leaders, and guideline developers seeking to optimize risk-based prevention strategies.

## 2. Materials and Methods

### 2.1. Study Population: Inclusion and Exclusion Criteria

Setting and data source: We analyzed participants from the Lithuanian High Cardiovascular Risk primary prevention program (LitHiR), a government-sponsored initiative launched in 2006 to reduce cardiovascular (CV) risk among middle-aged adults in Lithuania. All assessments for the present study were performed at a single tertiary center, Vilnius University Hospital Santaros Klinikos (Vilnius, Lithuania), using standardized prospectively collected data spanning 2006–2023.

Target population and eligibility: The program targeted adults aged 40–65 years without clinically evident cardiovascular disease but with elevated cardiometabolic risk, as defined by metabolic syndrome (MetS). For this analysis, we included consecutive LitHiR participants within this age range who met the MetS definition and had sufficiently complete data to support risk estimation and eligibility classification across the included models.

Definition of metabolic syndrome: MetS was defined according to the updated National Cholesterol Education Program Adult Treatment Panel III (NCEP ATP III) criteria [14], requiring ≥3 of the following:

1. Blood pressure: Systolic (SBP) ≥ 130 mmHg, diastolic (DBP) ≥85 mmHg, or a documented diagnosis of hypertension;
2. Central adiposity: Waist circumference ≥ 88 cm (women) or ≥102 cm (men);
3. High-density lipoprotein cholesterol (HDL-C): <1.29 mmol/L (women) or <1.03 mmol/L (men);
4. Triglycerides: ≥1.7 mmol/L or on pharmacotherapy to lower triglycerides;
5. Glycemia: Diagnosed type 2 diabetes mellitus or fasting plasma glucose ≥ 5.6 mmol/L.

Data elements and completeness: The analytic dataset was derived from LitHiR’s uniform electronic records and included variables required for risk calculation and cohort description: LDL-C, HDL-C, total cholesterol, SBP, DBP, fasting glucose, creatinine, C-reactive protein (CRP), the urine albumin-to-creatinine ratio, and family and medication history. Participants lacking key measurements required for risk estimation were excluded.

Exclusion criteria: We excluded individuals with conditions likely to confound risk assessment or indicate established CVD or severe systemic diseases, including silent myocardial ischemia, coronary artery disease, transient ischemic attack, peripheral arterial disease, ischemic or hemorrhagic stroke, oncologic disease, chronic or persistent arrhythmias, severe renal or hepatic dysfunction, major psychiatric illness, gout, pregnancy, current xanthine oxidase inhibitor therapy, or active drug addiction.

Analytic cohort size: After applying the eligibility, exclusion, and completeness criteria, the final analytic cohort comprised 11,174 participants aged 40–65 years.

### 2.2. Risk Prediction Models

#### 2.2.1. Systematic Coronary Risk Evaluation 2 (SCORE2)

SCORE2 estimates the 10-year risk of first fatal or nonfatal cardiovascular disease in Europe among adults aged 40–69 years without prior CVD, representing an update of the original SCORE with contemporary data [4]. Predictors include age, sex, systolic blood pressure, non-HDL cholesterol, and smoking status. Risk was calculated using the official calculator (https://u-prevent.com/calculators/score2; accessed on 2 June 2023). For this study, Lithuania was classified according to the SCORE2 country-risk stratification as a very-high-risk European region; therefore, the very-high-risk regional recalibration was applied in accordance with the 2021 ESC guideline framework. The computation procedure itself was unmodified.

#### 2.2.2. Predicting Risk of Cardiovascular Disease EVENTs (PREVENT)

PREVENT provides 10-year risk estimates for ages 30–79 years, and it was developed by the American Heart Association Cardiovascular-Kidney-Metabolic Scientific Advisory Group using > 6 million individuals [6,7]. We computed the 10-year CVD risk with the AHA tool (https://professional.heart.org/en/guidelines-and-statements/prevent-calculator; accessed on 1 March 2024). ZIP code entry was not applicable and was omitted; otherwise, the default implementation was used.

#### 2.2.3. Pooled Cohort Equations (PCEs)

PCEs estimate the 10-year ASCVD risk for African American and non-Hispanic White adults aged 40–79 years, integrating age, sex, race, systolic blood pressure, antihypertensive treatment, total cholesterol, HDL-cholesterol, diabetes, and smoking [5]. Calculations followed the ACC/AHA web application (https://static.heart.org/riskcalc/app/index.html#!/baseline-risk; accessed on 1 June 2023) without modification.

#### 2.2.4. Multi-Ethnic Study of Atherosclerosis (MESA) Risk Score

The MESA score estimates the 10-year risk of CHD/CVD events in adults aged 45–85 years free of clinical CVD, and it is derived from a multi-ethnic cohort [11]. We used the official calculator (https://www.mesa-nhlbi.org/MESACHDRisk/MesaRiskScore/RiskScore.aspx; accessed on 2 June 2023). Coronary artery calcium (CAC) data was not included because CAC information was unavailable in our dataset; the remaining inputs followed the standard algorithm.

#### 2.2.5. QRISK3 Risk Calculator (QRISK3)

QRISK3 (2017) updates QRISK2 to predict the 10-year CVD risk in the UK for ages 25–84 years by adding risk factors such as corticosteroid use, migraine, atypical antipsychotic therapy, systemic lupus erythematosus, severe mental illness, blood pressure variability, and erectile dysfunction, alongside conventional predictors [8]. Risk was computed using the official interface (https://qrisk.org/; accessed on 2 June 2023) with default settings.

#### 2.2.6. Assessing Cardiovascular Risk Using SIGN (ASSIGN)

ASSIGN estimates the 10-year CVD risk in individuals without prior CVD, incorporating social deprivation (Scottish Index of Multiple Deprivation) and family history in addition to standard risk factors [9]. We used the ASSIGN calculator (https://www.rightdecisions.scot.nhs.uk/; accessed on 2 June 2023). The deprivation index component was not applicable in our setting and was omitted; otherwise, the default computation was retained.

#### 2.2.7. Australian CVD Risk Score (AusCVDRisk)

AusCVDRisk predicts the 5-year CVD risk for adults aged 30–79 years without established CVD and those not meeting the automatic high-risk criteria. It is based on the New Zealand PREDICT-1 equation and recalibrated for Australia [10]. We used the national calculator (https://www.cvdcheck.org.au/calculator; accessed on 2 August 2023). Postcode entry was not applicable and was omitted; the rest of the computation followed the published implementation.

#### 2.2.8. Framingham Risk Score for Hard Coronary Heart Disease (FRS-hCHD)

FRS-hCHD estimates the 10-year risk of hard CHD (coronary death or myocardial infarction) in non-diabetic adults aged 30–79 years without prior CHD or claudication using data on age, sex, total cholesterol, HDL-cholesterol, systolic blood pressure, smoking, and antihypertensive therapy [14]. We used the MDCalc implementation (https://www.mdcalc.com/calc/38/framingham-risk-score-hard-coronary-heart-disease; accessed on 1 June 2023) without modifications.

#### 2.2.9. Reynolds Risk Score (RRS)

RRS predicts the 10-year risk of major cardiovascular events, incorporating conventional risk factors plus high-sensitivity C-reactive protein and family history of premature atherosclerosis; models exist for women and men [12]. We used the official calculator (http://www.reynoldsriskscore.org/; accessed on 2 June 2023) with standard inputs; no changes were made to the computation.

### 2.3. Variable Definitions

**Figure 1:**
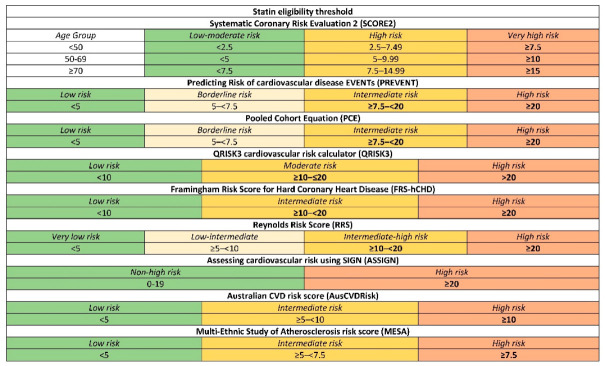
Presentation of statin eligibility thresholds among different prediction models. Variables in bold indicate eligibility for statin therapy. For each model, statin eligibility was defined according to the treatment threshold mapped to the corresponding guideline or model-specific decision framework used in the analysis: in the case of SCORE2, age-specific thresholds for very-high-risk European regions are applicable to Lithuania.

We evaluated nine CV RPMs with their guideline-recommended risk thresholds for statin initiation. An individual was considered “statin-eligible” under a given model if their calculated risk met or exceeded that model’s treatment threshold. This was therefore a decision-level comparison of complete risk-assessment frameworks, rather than a comparison of interchangeable numerical risk estimates. Each RPM was interpreted within its native context, including its original outcome definition, prediction horizon, calibration approach, and associated treatment threshold. Figure 1 graphically depicts these thresholds among the various models.

Risk scores were calculated using lipid and clinical values measured at the baseline LitHiR assessment. For participants already receiving lipid-lowering therapy at the time of assessment, measured on-treatment lipid values were used because untreated pre-treatment lipid values were not uniformly available in the registry. Information on baseline statin treatment was recorded and is reported in Table 1. When a model explicitly required information on lipid-lowering therapy as an input, this variable was entered according to the native calculator specification.

### 2.4. Statistical Analysis

#### 2.4.1. Agreement Metrics

Using the binary eligibility outcomes (yes/no) for each model, we computed multiple agreement statistics for every pair of models. Cohen’s kappa (κ) was used to assess chance-corrected agreement, while Gwet’s AC1 provided an alternative chance-corrected index less sensitive to prevalence imbalance. We also calculated Positive Percent Agreement (PPA) and Negative Percent Agreement (NPA)—the proportion of overlapping “eligible” classifications among all instances where at least one model was positive, and similarly for negatives—to contextualize agreement on positive vs. negative decisions. The Jaccard index (intersection over union of eligible sets) was computed as a direct measure of overlap in recommended patients. Additionally, McNemar’s chi-square test was applied to each model pair to detect systematic differences in overall statin assignment rates (i.e., whether one model tended to classify more patients than the other). Because Cohen’s κ may be affected by the marginal prevalence of binary classifications, particularly when one or both models classify very few individuals as eligible, κ was not interpreted in isolation but alongside AC1, PPA, NPA, Jaccard overlap, and McNemar testing. All metrics were derived for the entire cohort and within sex-specific subsets.

#### 2.4.2. Clustering and Consensus Analyses

To visualize patterns, we provided heatmaps of pairwise κ and Jaccard values. In addition, we examined “consensus” rules across models: for each participant we tallied how many of the nine models would recommend statin therapy, and then assessed what fraction of the population would be eligible if a given consensus threshold was applied. This analysis illustrates the impact of requiring agreement among multiple models—from at least one model (most inclusive) up to all models (most strict)—effectively mapping the consensus footprint of statin eligibility across the cohort. This consensus approach, stratified by sex, further highlights differences in how uniformly each group is identified by current risk models for preventive statin treatment.

Statistical analyses were conducted in Python 3.14.15. Agreement measures (Cohen’s κ, Gwet’s AC1, PPA, NPA, and Jaccard) were computed using custom Python routines based on 2 × 2 contingency tables (NumPy/pandas); McNemar’s tests were conducted with SciPy. Where applicable, Cohen’s κ was cross-checked with scikit-learn. The significance threshold was α = 0.05 (two-sided).

### 2.5. Ethical Considerations

This research was authorized by the Vilnius Regional Biomedical Research Ethics Committee (permission No. 2019/3-1104-603).

## 3. Results

### 3.1. Descriptive Statistics

Baseline characteristics of the study population are shown in Table 1.

### 3.2. Eligibility Rate Analysis

**Figure 2:**
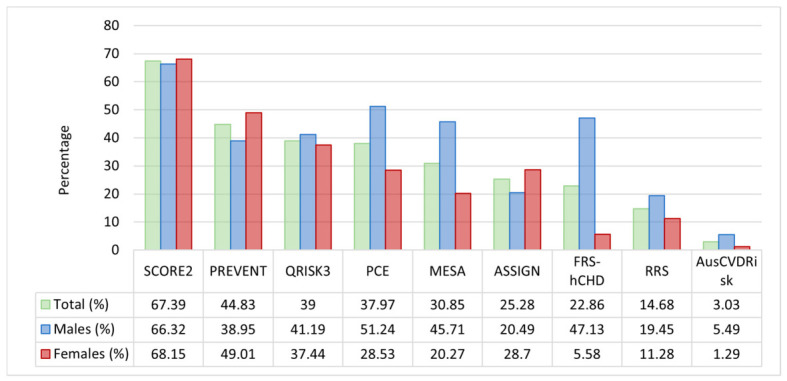
Distribution of statin eligibility across nine cardiovascular risk prediction models. Abbreviations: SCORE2, Systematic Coronary Risk Evaluation 2; PREVENT, Predicting Risk of Cardiovascular Disease EVENTs; PCE, Pooled Cohort Equation; MESA, Multi-Ethnic Study of Atherosclerosis; QRISK3, QRISK3 cardiovascular risk calculator; ASSIGN, Assessing Cardiovascular Risk Using SIGN; FRS-hCHD, Framingham Risk Score for Hard Coronary Heart Disease; RRS, Reynolds Risk Score; AusCVDRisk, Australian cardiovascular disease risk score.

In the statin eligibility analysis, substantial variability was observed across the nine cardiovascular risk prediction models in categorizing patients as eligible for statin therapy (Figure 2). In the overall cohort, the SCORE2 model identified the highest eligibility rate with 7530 patients (67.39%), whereas the AusCVDRisk model reported the lowest, classifying only 339 patients (3.03%) as eligible. Intermediate eligibility rates were noted for the other models: PREVENT identified 5009 patients (44.83%). PCE identified 4243 (37.97%). MESA revealed 3447 (30.85%). QRISK3 found 4358 (39%). FRS-hCHD identified 2554 (22.86%). RRS encompassed 1640 (14.68%), and ASSIGN had 2825 (25.28%).

When stratified by sex, distinct differences emerged. Among male subjects, SCORE2 again yielded the highest eligibility rate with 3082 males (66.32%), while PCE and MESA classified 2381 (51.24%) and 2124 (45.71%) males as eligible, respectively; in contrast, AusCVDRisk identified only 255 males (5.49%) as eligible. In female subjects, SCORE2 maintained a high eligibility rate with 4448 females (68.15%), and PREVENT classified 3199 females (49.01%) as eligible; however, FRS-hCHD demonstrated a striking difference between genders by identifying only 364 females (5.58%) as eligible compared to 2190 males (47.13%).

These findings underscore substantial heterogeneity in statin eligibility determination across different models. However, these differences should be interpreted as variation in guideline-based treatment classification rather than as evidence that any individual model is more accurate or clinically preferable. Their clinical relevance therefore lies primarily in demonstrating how model and threshold selection may affect the consistency of preventive treatment allocation.

### 3.3. Eligibility Overlap Analysis

**Figure 3:**
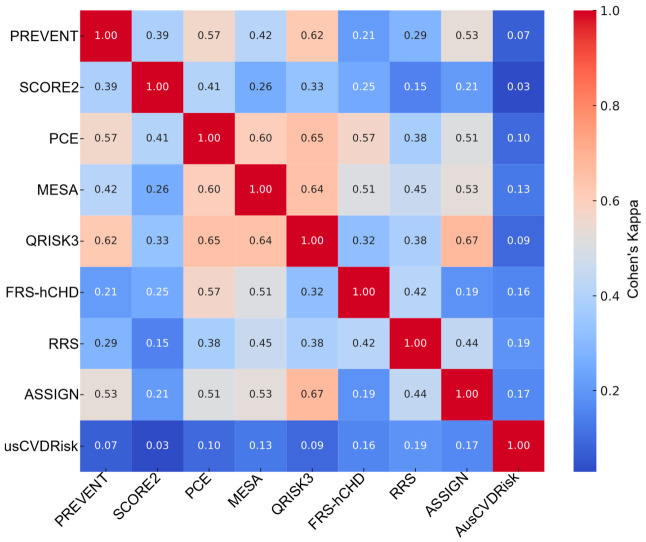
Heatmap (Cohen’s κ) illustrating pairwise statin eligibility overlap across nine cardiovascular risk prediction models. Agreement is expressed using Cohen’s κ, with higher values indicating stronger agreement between models. Model labels are shown on both axes, and the color scale represents the magnitude of κ. Abbreviations: SCORE2, Systematic Coronary Risk Evaluation 2; PREVENT, Predicting Risk of Cardiovascular Disease EVENTs; PCE, Pooled Cohort Equation; MESA, Multi-Ethnic Study of Atherosclerosis; QRISK3, QRISK3 cardiovascular risk calculator; ASSIGN, Assessing Cardiovascular Risk Using SIGN; FRS-hCHD, Framingham Risk Score for Hard Coronary Heart Disease; RRS, Reynolds Risk Score; AusCVDRisk, Australian cardiovascular disease risk score.

**Figure 4:**
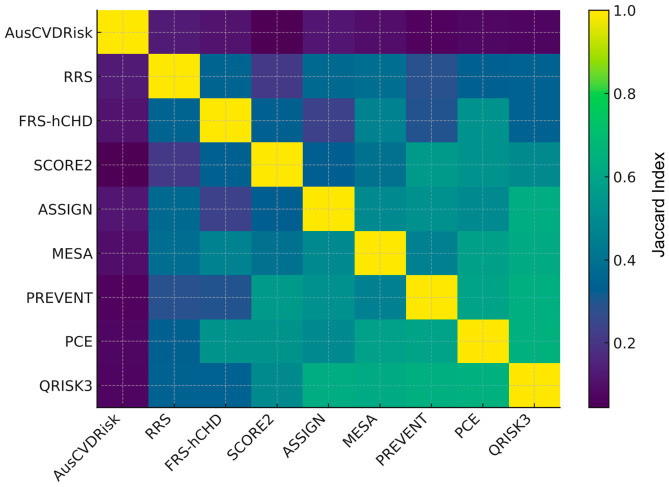
Heatmap of the Jaccard index for pairwise overlap of positive statin eligibility across nine cardiovascular risk prediction models. Abbreviations: SCORE2, Systematic Coronary Risk Evaluation 2; PREVENT, Predicting Risk of Cardiovascular Disease EVENTs; PCE, Pooled Cohort Equation; MESA, Multi-Ethnic Study of Atherosclerosis; QRISK3, QRISK3 cardiovascular risk calculator; ASSIGN, Assessing Cardiovascular Risk Using SIGN; FRS-hCHD, Framingham Risk Score for Hard Coronary Heart Disease; RRS, Reynolds Risk Score; AusCVDRisk, Australian cardiovascular disease risk score.

In the overall cohort, pairwise agreement between models ranged widely (Figure 3) and full pairwise statistics (κ, AC1, PPA/NPA, McNemar’s *p*, and Jaccard) for overall, males, and females are provided in Tables S1–S3. Cohen’s κ values extended from 0.03 for SCORE2 vs. AusCVDRisk to 0.67 for QRISK3 vs. ASSIGN, indicating minimal to moderate concordance across model pairings. Concordance was also high for PCE vs. QRISK3 (κ = 0.65) and MESA vs. QRISK3 (κ = 0.64), whereas PREVENT vs. AusCVDRisk exhibited low agreement (κ = 0.07). Median agreement across all pairs was κ_median = 0.38 (IQR 0.19–0.51). Gwet’s AC1, which is less sensitive to prevalence, yielded higher central tendency (AC1_median = 0.58, IQR 0.37–0.68) while preserving the same ranking: for instance, QRISK3–ASSIGN had AC1 = 0.74, PCE–QRISK3 had AC1 = 0.69, and MESA–QRISK3 had AC1 = 0.70, whereas SCORE2–AusCVDRisk showed AC1 = −0.18, reflecting extreme marginal imbalance. Agreement was consistently stronger for negatives than positives: NPA_median = 0.82 (IQR 0.72–0.87) vs. PPA_median = 0.53 (IQR 0.38–0.69). For exemplar pairs, PCE–QRISK3 had PPA = 0.78 and NPA = 0.87; in contrast, SCORE2–AusCVDRisk had PPA = 0.09 and NPA = 0.50. The Jaccard index echoed these patterns (Figure 4): PCE–QRISK3 Jaccard = 0.65 vs. SCORE2–AusCVDRisk Jaccard = 0.045. McNemar’s tests frequently indicated marginal asymmetry (e.g., PCE–QRISK3 *p* ≈ 0.008; many pairs *p* < 0.001), underscoring directional differences in which patients are labeled eligible.

**Figure 5:**
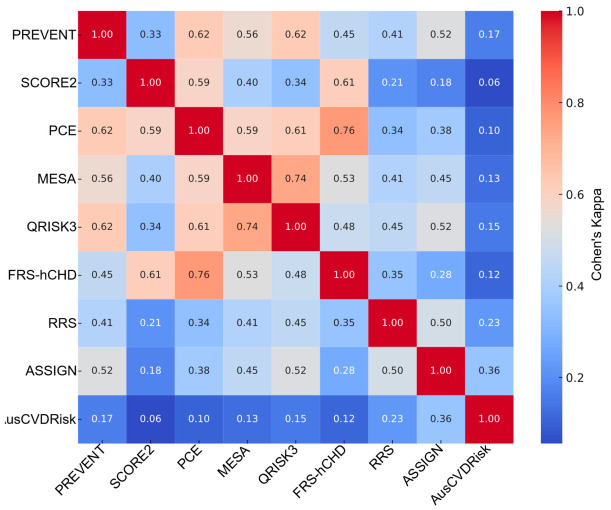
Heatmap (Cohen’s κ) illustrating pairwise statin eligibility overlap across nine cardiovascular risk prediction models among males. Agreement is expressed using Cohen’s κ, with higher values indicating stronger agreement between models. Model labels are shown on both axes, and the color scale represents the magnitude of κ. Abbreviations: SCORE2, Systematic Coronary Risk Evaluation 2; PREVENT, Predicting Risk of Cardiovascular Disease EVENTs; PCE, Pooled Cohort Equation; MESA, Multi-Ethnic Study of Atherosclerosis; QRISK3, QRISK3 cardiovascular risk calculator; ASSIGN, Assessing Cardiovascular Risk Using SIGN; FRS-hCHD, Framingham Risk Score for Hard Coronary Heart Disease; RRS, Reynolds Risk Score; AusCVDRisk, Australian cardiovascular disease risk score.

When stratified by sex, similar heterogeneity emerged with notable shifts in which pairs aligned most closely. Among males (Figure 5), PCE–FRS-hCHD showed the highest concordance (κ = 0.76, AC1 = 0.78, PPA = 0.82, NPA = 0.93, and Jaccard = 0.72), followed by MESA–QRISK3 (κ = 0.74, AC1 = 0.74, PPA = 0.78, NPA = 0.91, and Jaccard = 0.68); SCORE2–AusCVDRisk again demonstrated minimal agreement (κ = 0.06, AC1 = −0.13, PPA = 0.13, NPA = 0.53, and Jaccard = 0.07). Across all male pairs, central tendencies were κ_median = 0.41 (IQR 0.26–0.54), AC1_median = 0.52 (IQR 0.38–0.61), PPA_median = 0.60 (IQR 0.45–0.75), NPA_median = 0.78 (IQR 0.73–0.82), and Jaccard_median = 0.43 (IQR 0.29–0.60). These values indicate that, in men, high-κ pairs typically exhibit concordance on both positive and negative classifications, whereas low-κ pairs are driven by negative-class agreement with strong marginal asymmetry (McNemar *p* in all cases < 0.01).

**Figure 6:**
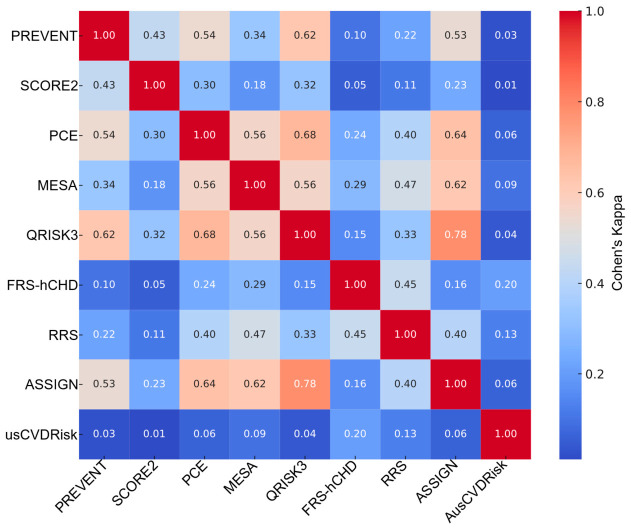
Heatmap (Cohen’s κ) illustrating pairwise statin eligibility overlap across nine cardiovascular risk prediction models among females. Agreement is expressed using Cohen’s κ, with higher values indicating stronger agreement between models. Model labels are shown on both axes, and the color scale represents the magnitude of κ. Abbreviations: SCORE2, Systematic Coronary Risk Evaluation 2; PREVENT, Predicting Risk of Cardiovascular Disease EVENTs; PCE, Pooled Cohort Equation; MESA, Multi-Ethnic Study of Atherosclerosis; QRISK3, QRISK3 cardiovascular risk calculator; ASSIGN, Assessing Cardiovascular Risk Using SIGN; FRS-hCHD, Framingham Risk Score for Hard Coronary Heart Disease; RRS, Reynolds Risk Score; AusCVDRisk, Australian cardiovascular disease risk score.

In females (Figure 6), the pattern differed; the top agreement was between QRISK3–ASSIGN (κ = 0.78, AC1 = 0.82, PPA = 0.85, NPA = 0.93, and Jaccard = 0.74), with PCE–QRISK3 also showing strong alignment (κ = 0.68, AC1 = 0.74, PPA = 0.78, NPA = 0.89, and Jaccard = 0.64). At the other extreme, SCORE2–AusCVDRisk in women exhibited virtually no agreement (κ = 0.01, AC1 = −0.22, PPA = 0.04, NPA = 0.49, and Jaccard = 0.019). Across all female pairs, the distribution was κ_median = 0.29 (IQR 0.13–0.48), AC1_median = 0.63 (IQR 0.32–0.74), PPA_median = 0.47 (IQR 0.21–0.67), NPA_median = 0.84 (IQR 0.69–0.89), and Jaccard_median = 0.31 (IQR 0.12–0.50). Thus, in women, even strongly agreeing pairs retain somewhat lower PPA than in men, and low-κ pairs are dominated by a lack of overlap on positives despite relatively high negative agreement; McNemar results again indicated prevalent marginal asymmetry (most *p* < 0.01).

Taken together, these findings show (i) a core of mutually concordant models (e.g., QRISK3, PCE, and MESA, with ASSIGN/FRS-hCHD joining in a sex-specific manner), (ii) a persistent outlier (AusCVDRisk) with minimal co-eligibility against most models, and (iii) a systematic imbalance wherein models agree more on non-eligibility than on eligibility. The alignment of model pairs—and the type of agreement (PPA vs. NPA)—differs by sex, implying that model choice can materially alter which women versus men are classified as candidates for statin therapy even when chance-corrected κ appears similar.

### 3.4. Consensus Analysis

**Figure 7:**
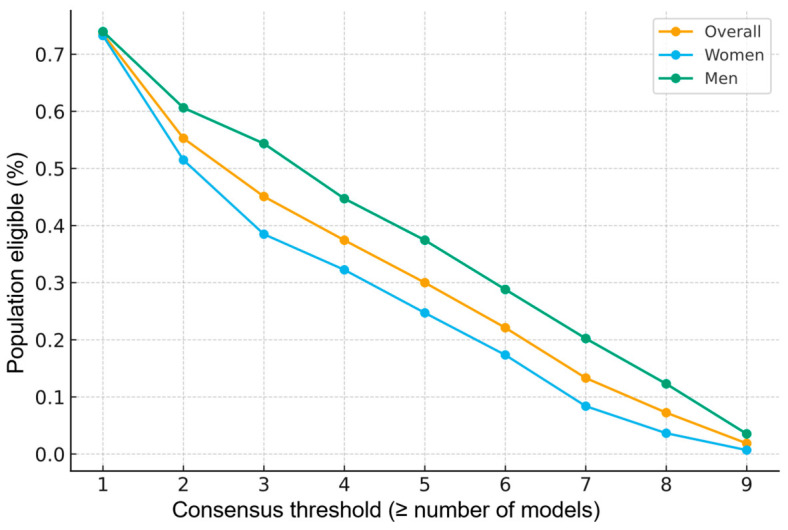
Statin eligibility consensus—proportion of individuals meeting the statin criteria under increasingly strict consensus requirements (number of risk prediction models).

We quantified the footprint of consensus policies of the form “eligible if ≥ k models recommend” across the nine risk prediction models, both overall and by gender (Figure 7; Table S4).

In the overall cohort, eligibility declined monotonically with increasing stringency. Coverage was 73.5% at k = 1, 55.3% at k = 2, and 45.1% at k = 3—with the latter being the nearest to 50% coverage (k closest to the “half-coverage” point). Stricter requirements further reduced eligibility to 30.0% at k = 5 and 1.87% at k = 9 (unanimity). The steepest drop occurred between k = 1 → 3 (−28.4 percentage points), indicating that much of the inter-model disagreement lies in the subset flagged by only one or two models.

When stratified by sex, the consensus curves separated meaningfully (Figure 7; Table S4). At the half-coverage vicinity (k = 3), eligibility was 54.4% in men vs. 38.5% in women (sex gap: −15.9 percentage points). This maximal sex gap also occurred at k = 3, suggesting that majority-style rules amplify sex differences in selection around intermediate stringency. At the extremes, k = 1 yielded high coverage in both sexes (reflecting “any-model” inclusivity), whereas k = 9 converged to very low coverage in both sexes (stringent unanimity), though still slightly higher in men, consistent with the greater cross-model concordance observed in the agreement analysis.

## 4. Discussion

### 4.1. Statin Eligibility Variability Across Models

Our findings demonstrate marked model-dependence in statin eligibility assignment. In the same Lithuanian primary-prevention cohort, the proportion classified as eligible varied more than twenty-fold, from 67% with SCORE2 to 3% with AusCVDRisk, while several other models occupied an intermediate range, including PCE (38%) and QRISK3 (~39%). This heterogeneity persisted after sex stratification. SCORE2 continued to classify most men and women as eligible (≈66–68%), whereas other models showed more pronounced sex-related variation; the clearest example was FRS-hCHD, which identified 47% of men but only ~6% of women as eligible. Together, these results indicate that statin eligibility is not a fixed clinical property, but rather an output strongly contingent on the selected risk framework. Similar disparities in “treat” versus “do-not-treat” classification across international algorithms have been described previously, with concordance varying substantially by the tool applied [15].

This dependence on model choice was further reflected in the modest inter-model concordance of binary eligibility decisions. Cohen’s κ ranged from near-zero agreement (κ ≈ 0.03 for SCORE2 vs. AusCVDRisk) to moderate agreement (κ ≈ 0.65–0.67 for PCE vs. QRISK3 and QRISK3 vs. ASSIGN), with a median κ of approximately 0.38 (IQR 0.19–0.51). Although Gwet’s AC1 yielded higher absolute values overall (median ≈ 0.58), it reproduced the same similarity structure, including extreme discordance for SCORE2 vs. AusCVDRisk (AC1 = −0.18) and relatively strong alignment for PCE vs. QRISK3 (AC1 ≈ 0.69). Agreement was consistently stronger for non-eligibility than for eligibility (NPA_median ≈ 0.82 vs. PPA_median ≈ 0.53), suggesting that the models shared a broader low-risk core but differed substantially in where they placed the treatment boundary. The Jaccard index supported the same interpretation, showing substantial overlap for PCE vs. QRISK3 (≈0.65) but minimal overlap for SCORE2 vs. AusCVDRisk (≈0.045). Frequent McNemar significance further indicated that these discrepancies reflected systematic directional differences in treatment assignment rather than random variation. Overall, these findings show limited cross-model consensus regarding who would receive statins under guideline-mapped thresholds, underscoring that model selection can materially shape preventive pharmacotherapy decisions in this cohort.

### 4.2. Drivers of Between-Model Discordance

Several intrinsic features of the RPMs likely explain the observed discordance. First, the models differ in predictor composition and derivation populations. Some incorporate additional nontraditional determinants of risk; for example, QRISK3 includes chronic conditions, corticosteroid use, and social deprivation, whereas RRS incorporates high-sensitivity C-reactive protein and family history. Such variables may increase the estimated risk in individuals who would remain below the threshold in more parsimonious models. By contrast, older tools such as FRS-hCHD rely on a narrower predictor set and were calibrated in historical cohorts, potentially yielding lower absolute risk estimates in groups that experienced fewer recorded events in those datasets, including women and younger adults [16].

Second, the models target different outcomes and time horizons. AusCVDRisk estimates the 5-year cardiovascular risk, whereas most other models estimate the 10-year risk; this shorter horizon would be expected to classify fewer individuals above treatment thresholds, consistent with its very low eligibility rate in our study (~3%) [17]. SCORE2 and PCE both estimate the 10-year risk of broader ASCVD-related outcomes, including nonfatal events, but differ in both risk formulation and treatment thresholds. SCORE2 was developed for European populations and uses age-specific thresholds in the 2021 ESC guidelines (≥7.5% at age 40–49 and ≥10% at age 50–69) [4], whereas PCE, as used in U.S. guidance, generally applies a fixed ≥7.5% 10-year ASCVD threshold for adults aged 40–75. These differences in policy architecture alone can produce discordant treatment recommendations for the same individual. Our analysis intentionally preserved each model’s guideline-endorsed threshold rather than harmonizing all models to a common absolute-risk cut-point because this better reflects the decisions encountered in real clinical practice. Thus, an individual may be eligible under one framework and not another even when estimated risks are broadly similar [3,4].

Third, discordance also arises from differences in endpoint definition and calibration. FRS-hCHD focuses on hard coronary events and excludes stroke and other vascular outcomes; accordingly, individuals whose risk burden is driven more by cerebrovascular events may receive lower estimates than under broader models such as QRISK3 or SCORE2. More generally, models predicting total fatal and nonfatal CVD can assign a higher absolute risk than those restricted to narrower coronary endpoints [18]. Calibration to different populations and eras adds further divergence. In external European analyses, both PCE and QRISK3 have been reported to overestimate contemporary event rates relative to the observed risk, with the mean predicted 10-year risk exceeding that of SCORE2 in the same cohort [2]. In such an external study of 66,000 individuals, the mean 10-year risk predicted by QRISK3 (7.4%) and PCE (6.4%) was substantially higher than that by SCORE2 (4.2%) in the same group [2]. Moreover, independent evaluation of QRISK3 in UK Biobank demonstrated systematic overprediction (up to ~20% in older participants) with age-dependent declines in discrimination, underscoring how conveyance into different sampling frames and event-ascertainment contexts can materially alter risk gradients and downstream eligibility [19].

The sex-stratified findings should also be interpreted in the context of baseline age imbalance and sex-specific model architecture. In the present cohort, women were substantially older than men, which would generally be expected to increase the estimated absolute risk in most RPMs. However, eligibility did not increase uniformly across models, indicating that age alone cannot explain the observed sex-specific patterns. These differences likely reflect the combined influence of biological sex, age distribution, sex-specific coefficients, model derivation cohorts, endpoint selection, and treatment-threshold architecture. For example, the markedly lower eligibility of women under FRS-hCHD may partly reflect the model’s focus on hard coronary outcomes and its historical derivation context, whereas broader CVD endpoints and contemporary sex-specific modeling may yield different classification patterns. Therefore, the observed sex differences should be interpreted as framework-dependent eligibility differences rather than direct evidence of true biological risk differences.

Consequently, more people cross the “high-risk” threshold under PCE or QRISK3 than under SCORE2 when applied to a low-risk European population; however, in our high-risk Lithuanian cohort (where event rates may be higher than those in Western Europe), SCORE2 turned out to flag a majority, but it still stood out in how it categorized risk versus its peers—consistent with reports indicating that SCORE2 often classifies more people as “high-risk” (especially in high-incidence regions) but conversely can drastically restrict eligibility in lower-risk settings [20]. Contemporary external validation work also suggests that SCORE2 performance is not uniform across subgroups: in EPIC-Norfolk, SCORE2 showed fair discrimination (AUC ~0.75) and overall calibration near unity, but with sex-directional calibration (underestimation in men and overestimation in women) [21] and in a Dutch population-based validation study, SCORE2 underpredicted risk, particularly in low socioeconomic and Surinamese-origin subgroups (overall observed-to-expected-ratio: ~1.2–1.3; Surinamese: ~1.9, rising further in low-socioeconomic strata) [22].

The PREVENT model, a newer tool incorporating additional factors, illustrated how model formulation can lead to intermediate behavior: it showed good concordance with PCE and QRISK3 (κ ~0.55) yet was “at odds” with SCORE2 (negative agreement, κ ≈ –0.09 in one analysis) [16]. At the same time, PREVENT was derived and validated using large contemporary datasets and was designed to be sex-specific and race-free, with kidney function and optional social-deprivation inputs explicitly considered [6]. Early implementation analyses suggest that replacing PCE with PREVENT can significantly reduce estimated risk and, at fixed thresholds, substantially reduce statin eligibility—effects that appear particularly pronounced in women and younger adults—thereby reinforcing the centrality of model choice in downstream treatment allocation [23].

Finally, it is also important to emphasize that our comparison was based on each model’s native, guideline-mapped threshold rather than on a harmonized absolute-risk metric. In other words, we asked whether a participant would qualify for statin therapy under model-specific guidance, not whether different models assigned the same numerical risk. This design necessarily incorporates threshold policy into the observed discordance. Models such as AusCVDRisk or FRS-hCHD are linked to different treatment cut-points than PCE or QRISK3, which amplifies disagreement in binary eligibility. Harmonizing thresholds might have increased apparent agreement, but such an approach would not correspond to actual clinical guideline use. From a practical and policy perspective, the relevant issue is precisely that different accepted frameworks can lead to opposing treatment decisions for the same person. Our results therefore support a cautious, model-aware interpretation of estimated cardiovascular risk and reinforce that model selection is not methodologically neutral but has direct implications for preventive treatment allocation. More broadly, the observed heterogeneity—arising from differences in predictors, endpoints, horizons, and threshold philosophies—highlights the need for continued evaluation, recalibration, and possibly greater harmonization of risk assessment strategies in preventive cardiology.

### 4.3. Study Strengths and Limitations

This study has several strengths. It was based on a large, well-characterized real-world primary-prevention cohort (*n* = 11,174) drawn from a structured national program, enabling direct comparison of nine widely used cardiovascular RPMs within the same clinical dataset and permitting sex-stratified analyses. In addition, the analytic framework extended beyond a single concordance metric by integrating complementary measures of agreement (Cohen’s κ, Gwet’s AC1, PPA/NPA, Jaccard overlap, and McNemar testing) together with a consensus-footprint analysis, thereby providing a more decision-oriented view of how eligibility changes under alternative operational rules.

This study also has important limitations. Its single-center design and restriction to adults with metabolic syndrome may limit generalizability to broader Lithuanian or international primary-prevention populations, particularly to lower-risk individuals, younger or older age groups, and populations without metabolic syndrome. The analysis focused on statin eligibility rather than observed cardiovascular outcomes; therefore, it does not permit conclusions regarding comparative predictive performance, calibration, discrimination, or clinical benefit. We did not report AUC/C-statistics, calibration plots, calibration slopes, or observed-to-expected event ratios because these metrics belong to external validation analysis, which addresses a distinct research question from the present decision-level agreement study. Such outcome-based validation in the LitHiR cohort is being addressed separately and is beyond the scope and length constraints of the present manuscript. Several models additionally required predictors unavailable in routine data, including CAC for MESA and deprivation- or postcode/ZIP-linked variables for ASSIGN, AusCVDRisk, and PREVENT; omission or default handling of these inputs may have introduced differential misclassification and reduced transportability. Finally, because the comparison intentionally applied guideline-mapped thresholds across models with heterogeneous prediction horizons and endpoints, part of the observed discordance likely reflects differing policy definitions of “high risk” rather than differences in latent risk ranking alone.

Importantly, the present analysis was not designed as an external validation study and therefore cannot determine which RPM most accurately predicts observed cardiovascular events in this Lithuanian cohort. The observed discordance reflects a composite of model structure, predictor selection, outcome definition, prediction horizon, regional calibration, and guideline-specific treatment thresholds. Accordingly, differences in statin eligibility should not be interpreted as intrinsic deficiencies of particular scores, but rather as evidence that currently accepted risk-based frameworks may operationalize preventive treatment eligibility differently. External validation with observed outcomes would be necessary to determine whether these eligibility differences correspond to differences in calibration, discrimination, clinical benefit, or overtreatment/undertreatment.

## 5. Conclusions

In this large Lithuanian primary-prevention cohort with metabolic syndrome, statin eligibility proved highly model-dependent when nine commonly used risk prediction models were applied with their guideline-mapped thresholds. Eligibility varied by more than twenty-fold, and inter-model agreement was generally modest, with substantially stronger concordance for non-eligibility than for eligibility. A subset of models (e.g., PCE, QRISK3, MESA, and ASSIGN) showed comparatively greater overlap, whereas SCORE2 and AusCVDRisk frequently diverged, often identifying very different eligible populations. Sex-stratified analyses and consensus rules further demonstrated that both the extent and the “direction” of disagreement depended on sex and on how many models were required to concur. Overall, these results indicate that selection of the risk-assessment framework—including the model, endpoint, prediction horizon, calibration, and embedded treatment threshold—can meaningfully change which patients are deemed statin-eligible in routine primary prevention. However, because this study assessed eligibility concordance rather than observed cardiovascular outcomes, the findings should not be interpreted as demonstrating the superiority or inferiority of any individual model. Further external validation in Lithuanian and comparable high-risk European populations is needed to determine the predictive accuracy and clinical consequences of these differences.

## Figures and Tables

**Table 1:** Baseline characteristics of the study cohort (*n* = 11,174).

	Characteristics			*p*-Value
	Total	Female 6527 (58.41)	Male 4647 (41.59)	<0.001
Age, years: mean (SD)	53.49 (6.47)	57.62 (4.21)	47.69 (4.27)	<0.001
Body mass index, kg/m^2^: mean (SD)	31.47 (4.18)	31.76 (4.68)	31.24 (3.85)	<0.001
Systolic blood pressure, mmHg: mean (SD)	137.16 (15.40)	137.15 (15.96)	137.17 (14.59)	0.939
Diastolic blood pressure, mmHg: mean (SD)	82.99 (10.69)	80.99 (10.38)	85.79 (10.48)	<0.001
Total cholesterol, mmol/L: mean (SD)	6.17 (1.37)	6.33 (1.40)	5.96 (1.31)	<0.001
Triglycerides, mmol/L: mean (SD)	2.11 (1.5)	1.88 (1.15)	2.43 (1.84)	<0.001
Low-density lipoprotein cholesterol, mmol/L: mean (SD)	3.98 (1.21)	4.13 (1.23)	3.76 (1.14)	<0.001
High-density lipoprotein cholesterol, mmol/L: mean (SD)	1.23 (0.31)	1.33 (0.31)	1.09 (0.26)	<0.001
Estimated glomerular filtration rate, ml/min/1.73 m^2^: mean (SD)	92.44 (11.83)	88.97 (10.74)	97.32 (11.56)	<0.001
C-reactive protein, mg/L: mean (SD)	2.81 (3.18)	3.09 (3.98)	2.56 (3.06)	<0.001
Creatinine, µmol/L: mean (SD)	71.69 (12.79)	65.60 (8.93)	80.25 (12.48)	<0.001
Fasting glucose, mmol/L: mean (SD)	6.31 (1.49)	6.30 (1.51)	6.32 (1.45)	0.463
Diabetes mellitus: *n* (%)	2063 (18.46)	1325 (20.3)	738 (15.89)	<0.001
Dyslipidemia treatment (statins): *n* (%)	1248 (11.17)	764 (11.7)	484 (10.42)	0.033
Hypertension treatment: *n* (%)	2939 (26.3)	1861 (28.5)	1078 (23.2)	<0.001
Antiplatelet treatment: *n* (%)	30 (0.27)	19 (0.29)	11 (0.24)	0.584
Current smoker: *n* (%)	2305 (20.63)	841 (12.9)	1464 (31.5)	<0.001
Ex-smoker: *n* (%)	686 (6.14)	189 (2.9)	497 (10.7)	<0.001

SD—standard deviation.

## Data Availability

The data underlying this article cannot be shared publicly due to the stipulations of the bioethical approval which precludes unrestricted data dissemination and confines access to individuals delineated within the approval framework. The data will be shared on reasonable request to the corresponding author.

## Supplementary Materials

The following supporting information can be downloaded at https://www.mdpi.com/article/10.3390/medicina62050979/s1, Table S1: Pairwise agreement for statin eligibility across nine cardiovascular risk models in the overall cohort (Cohen’s κ, Gwet’s AC1, PPA, NPA, Jaccard, and McNemar’s *p*); Table S2: Pairwise agreement for statin eligibility across nine cardiovascular risk models among males (Cohen’s κ, Gwet’s AC1, PPA, NPA, Jaccard, and McNemar’s *p*); Table S3: Pairwise agreement for statin eligibility across nine cardiovascular risk models among females (Cohen’s κ, Gwet’s AC1, PPA, NPA, Jaccard, and McNemar’s *p*); Table S4: Consensus statin eligibility by consensus threshold k (overall cohort and stratified by sex). Proportions and counts eligible under the rule “eligible if ≥k models recommend” for k = 1–9 include overall, women, and men.

## Abbreviations

The following abbreviations are used in this manuscript:

ACC	American College of Cardiology
AC1	Gwet’s Agreement Coefficient 1
AHA	American Heart Association
ASCVD	Atherosclerotic cardiovascular disease
ASSIGN	Assessing Cardiovascular Risk Using SIGN
AUC	Area under the curve
AusCVDRisk	Australian cardiovascular disease risk score
BMI	Body mass index
CAC	Coronary artery calcium
CHD	Coronary heart disease
CRP	C-reactive protein
CV	Cardiovascular
CVD	Cardiovascular disease
DBP	Diastolic blood pressure
ESC	European Society of Cardiology
FRS-hCHD	Framingham Risk Score for Hard Coronary Heart Disease
HDL-C	High-density lipoprotein cholesterol
IQR	Interquartile range
LDL-C	Low-density lipoprotein cholesterol
LitHiR	Lithuanian High Cardiovascular Risk primary prevention program
McNemar’s *p*	*p*-value from McNemar’s test
MESA	Multi-Ethnic Study of Atherosclerosis
MetS	Metabolic syndrome
NCEP ATP III	National Cholesterol Education Program Adult Treatment Panel III
NPA	Negative Percent Agreement
PCE	Pooled Cohort Equation
PCSK9	Proprotein convertase subtilisin/kexin type 9
PPA	Positive Percent Agreement
PREVENT	Predicting Risk of cardiovascular disease EVENTs
QRISK3	QRISK3 cardiovascular risk calculator
RPMs	Risk prediction models
RRS	Reynolds Risk Score
SBP	Systolic blood pressure
SCORE2	Systematic Coronary Risk Evaluation 2
SD	Standard deviation
SIGN	Scottish Intercollegiate Guidelines Network
UK	United Kingdom
US	United States
ZIP	Zone Improvement Plan
κ	Cohen’s kappa
