# Supplementary material for: The Role of Cardiovascular Risk Prediction Model Selection in Primary Prevention: An Observational Study of Statin Eligibility Agreement Across Nine Scores in a Lithuanian Primary-Prevention Cohort
Source: Medicina (Kaunas). 2026 May 17;62(5):979. doi: 10.3390/medicina62050979 (PMC13208909; doi:10.3390/medicina62050979)
Supplement: Supplementary file 1 [file medicina-62-00979-s001.zip › medicina-4300318-supplementary.pdf]

Table S1. Pairwise agreement for statin eligibility across nine cardiovascular risk models in the overall cohort (Cohen's κ, Gwet's AC1, PPA, NPA, Jaccard, McNemar's *p*).

Model—A	Model—B	Cohen's κ	Gwet's AC1	PPA	NPA	Jaccard index	McNemar's *p*-value
ASSIGN	QRISK3	0.671	0.740	0.772	0.892	0.629	<0.001
PCE	QRISK3	0.650	0.685	0.785	0.865	0.645	0.008
MESA	QRISK3	0.644	0.701	0.767	0.875	0.622	<0.001
PREVENT	QRISK3	0.621	0.639	0.779	0.841	0.638	<0.001
MESA	PCE	0.602	0.670	0.737	0.862	0.584	<0.001
FRS-hCHD	PCE	0.571	0.676	0.693	0.866	0.531	<0.001
PREVENT	PCE	0.567	0.590	0.745	0.820	0.594	<0.001
PREVENT	ASSIGN	0.534	0.594	0.685	0.830	0.521	<0.001
ASSIGN	MESA	0.528	0.679	0.659	0.867	0.492	<0.001
ASSIGN	PCE	0.508	0.618	0.658	0.842	0.490	<0.001
FRS-hCHD	MESA	0.506	0.678	0.636	0.866	0.466	<0.001
MESA	RRS	0.454	0.693	0.563	0.871	0.391	<0.001
ASSIGN	RRS	0.438	0.731	0.542	0.886	0.372	<0.001
FRS-hCHD	RRS	0.416	0.741	0.520	0.889	0.352	<0.001
PREVENT	MESA	0.415	0.470	0.629	0.774	0.459	<0.001
SCORE2	PCE	0.408	0.361	0.696	0.661	0.533	<0.001
SCORE2	PREVENT	0.389	0.376	0.718	0.639	0.560	<0.001
QRISK3	RRS	0.383	0.571	0.515	0.822	0.347	<0.001
PCE	RRS	0.380	0.579	0.511	0.825	0.343	<0.001
SCORE2	QRISK3	0.331	0.282	0.661	0.615	0.494	<0.001
FRS-hCHD	QRISK3	0.317	0.475	0.514	0.782	0.346	<0.001
PREVENT	RRS	0.287	0.432	0.445	0.765	0.286	<0.001
SCORE2	MESA	0.260	0.161	0.573	0.588	0.402	<0.001
SCORE2	FRS-hCHD	0.246	0.112	0.504	0.592	0.337	<0.001
PREVENT	FRS-hCHD	0.214	0.329	0.452	0.720	0.292	<0.001
SCORE2	ASSIGN	0.211	0.080	0.501	0.569	0.334	<0.001
AusCVDRisk	RRS	0.193	0.838	0.233	0.926	0.132	<0.001
ASSIGN	FRS-hCHD	0.187	0.531	0.382	0.804	0.236	<0.001
ASSIGN	AusCVDRisk	0.166	0.705	0.211	0.870	0.118	<0.001
AusCVDRisk	FRS-hCHD	0.160	0.734	0.205	0.882	0.114	<0.001
SCORE2	RRS	0.151	-0.025	0.356	0.552	0.216	<0.001
AusCVDRisk	MESA	0.129	0.612	0.177	0.832	0.097	<0.001
AusCVDRisk	PCE	0.097	0.482	0.148	0.780	0.080	<0.001
AusCVDRisk	QRISK3	0.093	0.462	0.144	0.772	0.078	<0.001
PREVENT	AusCVDRisk	0.074	0.343	0.127	0.725	0.068	<0.001
SCORE2	AusCVDRisk	0.030	-0.184	0.086	0.503	0.045	<0.001

PPA—Positive Percent Agreement; NPA—Negative Percent Agreement.

Table S2. Pairwise agreement for statin eligibility across nine cardiovascular risk models among males (Cohen's κ, Gwet's AC1, PPA, NPA, Jaccard, McNemar's *p*).

Model—A	Model—B	Cohen's κ	Gwet's AC1	PPA	NPA	Jaccard index	McNemar's *p*-value
FRS-hCHD	PCE	0.765	0.765	0.880	0.884	0.786	<0.001
MESA	QRISK3	0.736	0.744	0.850	0.885	0.740	<0.001
PREVENT	QRISK3	0.624	0.652	0.774	0.849	0.632	<0.001
PREVENT	PCE	0.621	0.623	0.789	0.827	0.651	<0.001
PCE	QRISK3	0.611	0.612	0.789	0.819	0.651	<0.001
SCORE2	FRS-hCHD	0.611	0.611	0.825	0.771	0.703	<0.001
SCORE2	PCE	0.591	0.606	0.827	0.754	0.706	<0.001
MESA	PCE	0.590	0.589	0.788	0.801	0.650	<0.001
PREVENT	MESA	0.564	0.582	0.747	0.815	0.597	<0.001
FRS-hCHD	MESA	0.529	0.533	0.747	0.781	0.597	0.046
PREVENT	ASSIGN	0.523	0.644	0.651	0.852	0.483	<0.001
ASSIGN	QRISK3	0.519	0.624	0.650	0.844	0.482	<0.001
ASSIGN	RRS	0.505	0.767	0.603	0.901	0.432	0.077
FRS-hCHD	QRISK3	0.476	0.488	0.706	0.768	0.546	<0.001
PREVENT	FRS-hCHD	0.453	0.471	0.687	0.763	0.523	<0.001
ASSIGN	MESA	0.451	0.532	0.606	0.805	0.435	<0.001
QRISK3	RRS	0.447	0.573	0.593	0.823	0.422	<0.001
MESA	RRS	0.415	0.506	0.575	0.794	0.403	<0.001
PREVENT	RRS	0.415	0.569	0.567	0.821	0.395	<0.001
SCORE2	MESA	0.400	0.392	0.725	0.649	0.568	<0.001
ASSIGN	PCE	0.379	0.417	0.561	0.755	0.390	<0.001
ASSIGN	AusCVDRisk	0.359	0.803	0.414	0.913	0.261	<0.001
FRS-hCHD	RRS	0.353	0.439	0.531	0.766	0.362	<0.001
SCORE2	QRISK3	0.342	0.308	0.676	0.624	0.511	<0.001
PCE	RRS	0.337	0.381	0.524	0.740	0.355	<0.001
SCORE2	PREVENT	0.330	0.284	0.659	0.621	0.491	<0.001
ASSIGN	FRS-hCHD	0.277	0.368	0.484	0.736	0.319	<0.001
AusCVDRisk	RRS	0.225	0.774	0.292	0.899	0.171	<0.001
SCORE2	RRS	0.211	0.073	0.449	0.586	0.289	<0.001
SCORE2	ASSIGN	0.177	0.036	0.435	0.567	0.278	<0.001
PREVENT	AusCVDRisk	0.167	0.489	0.247	0.785	0.141	<0.001
AusCVDRisk	QRISK3	0.153	0.444	0.235	0.767	0.133	<0.001
AusCVDRisk	MESA	0.129	0.350	0.214	0.730	0.120	<0.001
AusCVDRisk	FRS-hCHD	0.115	0.314	0.202	0.715	0.112	<0.001
AusCVDRisk	PCE	0.105	0.229	0.193	0.681	0.107	<0.001
SCORE2	AusCVDRisk	0.057	-0.128	0.152	0.525	0.082	<0.001

PPA—Positive Percent Agreement; NPA—Negative Percent Agreement.

Table S3. Pairwise agreement for statin eligibility across nine cardiovascular risk models among females (Cohen's κ, Gwet's AC1, PPA, NPA, Jaccard, McNemar's *p*).

Model—A	Model—B	Cohen's κ	Gwet's AC1	PPA	NPA	Jaccard index	McNemar's *p*-value
ASSIGN	QRISK3	0.782	0.825	0.853	0.927	0.744	<0.001
PCE	QRISK3	0.675	0.740	0.780	0.892	0.640	<0.001
ASSIGN	PCE	0.641	0.752	0.744	0.897	0.592	0.722
PREVENT	QRISK3	0.622	0.631	0.783	0.834	0.643	<0.001
ASSIGN	MESA	0.621	0.775	0.711	0.906	0.551	<0.001
MESA	PCE	0.562	0.741	0.666	0.892	0.499	<0.001
MESA	QRISK3	0.562	0.684	0.677	0.869	0.512	<0.001
PREVENT	PCE	0.544	0.570	0.709	0.815	0.549	<0.001
PREVENT	ASSIGN	0.535	0.560	0.703	0.811	0.542	<0.001
MESA	RRS	0.468	0.804	0.545	0.915	0.374	<0.001
FRS-hCHD	RRS	0.448	0.898	0.489	0.953	0.324	<0.001
SCORE2	PREVENT	0.431	0.443	0.755	0.654	0.607	<0.001
ASSIGN	RRS	0.401	0.705	0.498	0.875	0.332	<0.001
PCE	RRS	0.397	0.704	0.494	0.874	0.328	<0.001
PREVENT	MESA	0.335	0.400	0.526	0.749	0.357	<0.001
QRISK3	RRS	0.329	0.572	0.445	0.821	0.286	<0.001
SCORE2	QRISK3	0.324	0.264	0.651	0.609	0.482	<0.001
SCORE2	PCE	0.300	0.192	0.582	0.609	0.410	<0.001
FRS-hCHD	MESA	0.289	0.784	0.351	0.904	0.213	<0.001
FRS-hCHD	PCE	0.238	0.671	0.309	0.858	0.183	<0.001
SCORE2	ASSIGN	0.234	0.117	0.544	0.572	0.373	<0.001
PREVENT	RRS	0.217	0.334	0.361	0.724	0.220	<0.001
AusCVDRisk	FRS-hCHD	0.202	0.943	0.219	0.972	0.123	<0.001
SCORE2	MESA	0.180	0.016	0.436	0.553	0.279	<0.001
ASSIGN	FRS-hCHD	0.160	0.636	0.239	0.843	0.136	<0.001
FRS-hCHD	QRISK3	0.151	0.502	0.234	0.790	0.132	<0.001
AusCVDRisk	RRS	0.131	0.879	0.151	0.943	0.082	<0.001
SCORE2	RRS	0.112	-0.091	0.284	0.528	0.165	<0.001
PREVENT	FRS-hCHD	0.099	0.266	0.189	0.696	0.104	<0.001
AusCVDRisk	MESA	0.093	0.764	0.115	0.893	0.061	<0.001
AusCVDRisk	PCE	0.063	0.635	0.086	0.840	0.045	<0.001
ASSIGN	AusCVDRisk	0.063	0.632	0.086	0.839	0.045	<0.001
SCORE2	FRS-hCHD	0.053	-0.171	0.151	0.504	0.082	<0.001
AusCVDRisk	QRISK3	0.043	0.474	0.066	0.776	0.034	<0.001
PREVENT	AusCVDRisk	0.027	0.235	0.051	0.681	0.026	<0.001
SCORE2	AusCVDRisk	0.012	-0.223	0.037	0.488	0.019	<0.001

PPA—Positive Percent Agreement; NPA—Negative Percent Agreement.

Table S4. Consensus statin-eligibility by consensus threshold k (overall cohort and stratified by sex). Proportions and counts eligible under the rule "eligible if ≥k models recommend" for k = 1–9; includes overall, women, and men, with a sex gap (women − men).

k	Overall—count	Overall—rate	Female—count	Female—rate	Male—count	Male—rate
1	8218	0.735	4781	0.732	3437	0.740
2	6177	0.553	3360	0.515	2817	0.606
3	5038	0.451	2512	0.385	2526	0.544
4	4183	0.374	2105	0.323	2078	0.447
5	3353	0.300	1613	0.247	1740	0.374
6	2471	0.221	1132	0.173	1339	0.288
7	1487	0.133	547	0.084	940	0.202
8	809	0.072	238	0.036	571	0.123
9	209	0.019	45	0.007	164	0.035

k denotes the number of risk models (of 9) required for eligibility. Rates are proportions of the respective stratum.
